# Largely Enhanced Saturable Absorption of a Complex of Plasmonic and Molecular-Like Au Nanocrystals

**DOI:** 10.1038/srep09735

**Published:** 2015-04-15

**Authors:** Si-Jing Ding, Fan Nan, Da-Jie Yang, Xiao-Li Liu, Ya-Lan Wang, Li Zhou, Zhong-Hua Hao, Qu-Quan Wang

**Affiliations:** 1 Department of Physics, Key Laboratory of Artificial Micro- and Nano-Structures of Ministry of Education, Wuhan University, Wuhan 430072, P. R. China; 2 Institute for Advanced Studies, Wuhan University, Wuhan 430072, P. R. China

## Abstract

A saturable absorber is a nonlinear functional material widely used in laser and photonic nanodevices. Metallic nanostructures have prominent saturable absorption (SA) at the plasmon resonance frequency owing to largely enhanced ground state absorption. However, the SA of plasmonic metal nanostructures is hampered by excited-state absorption processes at very high excitation power, which usually leads to a changeover from SA to reversed SA (SA→RSA). Here, we demonstrate tunable nonlinear absorption behaviours of a nanocomplex of plasmonic and molecular-like Au nanocrystals. The SA→RSA process is efficiently suppressed, and the stepwise SA→SA process is fulfilled owing to energy transfer in the nanocomplex. Our observations offer a strategy for preparation of the saturable absorber complex and have prospective applications in liquid lasers as well as one-photon nonlinear nanodevices.

Light-matter interaction in the strong excitation field regime usually exhibits nonlinear absorption behaviours. Two typical types of optical nonlinear absorbers exist and are known as saturable absorbers and optical limiters^1^,^2^,^3^,^4^,^5^,^6^,^7^,^8^,^9^,^10^,^11^,^12^,^13^,^14^,^15^,^16^,^17^,^18^,^19^,^20^. Interestingly, metal nanocrystals can be used as either saturable absorbers or optical limiters depending on their sizes, shapes, and plasmon resonance wavelengths and strengths^6^,^8^,^12^,^13^. For saturable absorbers^1^,^2^,^3^,^4^, the absorption coefficient decreases as the light intensity increases, and this property is commonly used to dynamically tune the *Q*-factor of the optical nonlinear devices by decreasing the losses at higher intensity. Saturable absorption (SA) is a one-photon nonlinear process and is induced by population bleaching of the ground state owing to an excited population that cannot relax to the ground state sufficiently fast at a large pump rate. For optical limiters with revised saturable absorption (RSA)^5^,^6^,^7^,^8^, the absorption coefficient increases as the light intensity increases, and these materials are widely used to protect from damage to the optical device at high power density.

The surface plasmon resonances of the metallic nanostructures strongly enhance the local electromagnetic field and have been widely used to enhance various light-matter interactions at the nanometer scale^21^,^22^,^23^,^24^,^25^,^26^,^27^,^28^,^29^,^30^,^31^,^32^,^33^. The plasmonic resonance wavelength and strength can be tuned by adjusting the size and shape of the metallic nanostructures^34^,^35^,^36^,^37^,^38^,^39^,^40^,^41^,^42^,^43^. The plasmonic metal nanostructures (especially Au and Ag nanocrystals) with large local fields enhance the one-photon SA by increasing the absorption cross section of the ground state, which also enhances the two-photon RSA by increasing the excited state absorption. The nonlinear SA→RSA changeover processes are observed in various plasmonic metal nanostructures^6^,^7^,^8^.

In recent years, it has been reported that the linear and nonlinear optical responses of ultra-small metal nanocrystals are strongly modulated by the quantum effect^8^,^44^,^45^. For instance, small Au nanocrystals (AuNCs) in the quantum size regime (with typical sizes of less than 2 nm) have an increased dielectric constant in the imaginary portion, which results in completely suppressed plasmon resonance and a molecular-like exciton absorption band edge. The molecular-like AuNCs in this quantum size regime have prominently weakened one- and two-photon nonlinear absorptions. The Au atomic clusters with sizes of less than 1.5 nm exhibit very large two-photon absorption owing to discrete levels of such quantum systems^8^,^46^. Therefore, a critical size exists for the smallest two-photon absorption, which indicates that the SA→RSA nonlinear processes could be suppressed in molecular-like AuNCs with an appropriate size^8^,^46^.

The coupling of plasmonic metal nanostructures generates much stronger local field enhancement. Furthermore, the strong plasmon-exciton interactions in a complex of metal nanostructures and semiconductor quantum dots or organic dye molecules have been extensively investigated because this structure induces intriguing behaviours, i.e., plasmon-molecule Rabi splitting, Fano resonance^47^,^48^,^49^,^50^,^51^, and plasmon resonance energy transfer^52^,^53^. However, nonlinear responses enhanced by the strong interaction of plasmonic and molecular-like AuNCs are seldom reported thus far.

In this paper, we investigate the nonlinear responses of a nanocomplex of plasmonic and molecular-like AuNCs. The individual plasmonic and molecular-like AuNCs with appropriate sizes exhibit SA→RSA and pure SA, respectively. The nanocomplex of plasmonic and molecular-like AuNCs demonstrates intriguing stepwise SA→SA processes with enhanced SA and suppressed RSA, which are explained by the energy transfer in the nanocomplex and provide a new strategy for tuning the one-photon nonlinear responses of plasmonic nanodevices.

## Results

### Linear Optical Responses of the Plasmonic and Molecular-Like AuNCs

**Figure 1:**
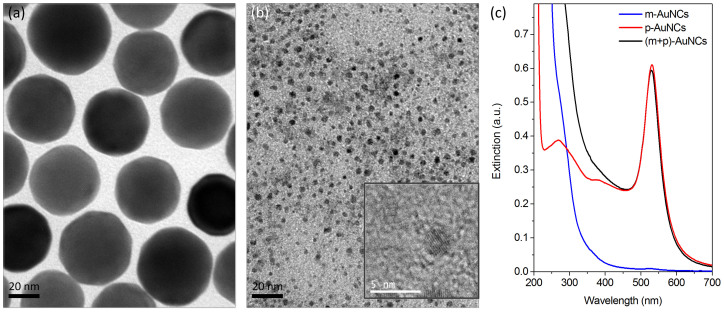
TEM images and extinction spectra of plasmonic and molecular-like AuNCs. (a) TEM image of the plasmonic AuNCs with an average diameter of ~50 nm. (b) TEM images showing that the average size of the molecular-like AuNCs is less than 2 nm. (c) Extinction spectra of the bare plasmonic (red line) and molecular-like (blue line) AuNCs and their mixtures (black line). The plasmon resonance wavelength of the plasmonic AuNCs is ~530 nm, and the absorption band edge of the molecular-like AuNCs is ~350 nm.

Figure 1 presents transmission electron microscopy (TEM) images and extinction spectra of plasmonic and molecular-like AuNCs. The plasmonic AuNCs have spherical shapes with an average size of ~50 nm (Figure 1a). The sizes of the molecular-like AuNCs are less than 2 nm (Figure 1b), and the high-resolution TEM (HRTEM) image in the inset clearly shows that the atomic distance of the AuNC is ~0.24 nm. The molecular-like AuNCs are generated from plasmonic AuNCs by thermal etching at a temperature of 190°C, which produces a mass of small AuNCs arranged around large structures and dispersed in aqueous suspensions (Figure S1). Both AuNCs have excellent photostability in aqueous suspensions during Z-scan measurements with laser irradiance of less than ~0.2 GW/cm^2^ (Figures S2, S3, and S6), which indicates that the fragmentation of plasmonic AuNCs and the formation of larger clusters from molecular-like AuNCs caused by the photo-thermal effect can be neglected at this low irradiance^1^,^54^,^55^,^56^. The resonance peak of the plasmonic AuNCs is located at ~528 nm (Figure 1c), which is nearly completely suppressed, and the band edge absorption at approximately 350 nm becomes prominent for the molecular-like AuNCs. The peak absorption intensity of the nanocomplex AuNCs is slightly smaller than the sum of the plasmonic and molecular-like structures, which indicates energy transfer between two types of AuNCs.

### Nonlinear Absorption of the Molecular-Like AuNCs: SA

**Figure 2:**
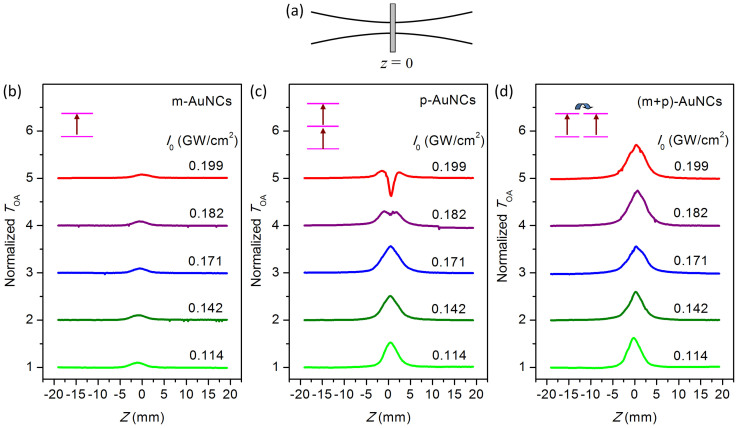
Z-scan nonlinear transmittance of individual and coupled plasmonic and molecular-like AuNCs. (a) Illustration of the geometry for the Z-scan measurement. (b) Laser-power-dependent Z-scan nonlinear transmittance of the molecular-like AuNCs. A peak in the Z-scan trace indicates a saturated absorption process of a two-level system (shown in inset). (c) Laser-power-dependent Z-scan nonlinear transmittance of the plasmonic AuNCs. The dip that appears in a broad peak in the Z-scan trace at high laser power indicates SA→RSA processes, and the RSA is induced by two-photon absorption process of a three-level system (shown in inset). (d) Laser-power-dependent Z-scan nonlinear transmittance of the coupled plasmonic and molecular-like AuNCs. A narrow peak folded on a broad peak in the Z-scan trace at high laser power indicates dual saturable absorptions (SA→SA processes) of two two-level systems (shown in inset). The Z-scan nonlinear transmittances with different laser powers are shifted vertically for clarity.

**Figure 3:**
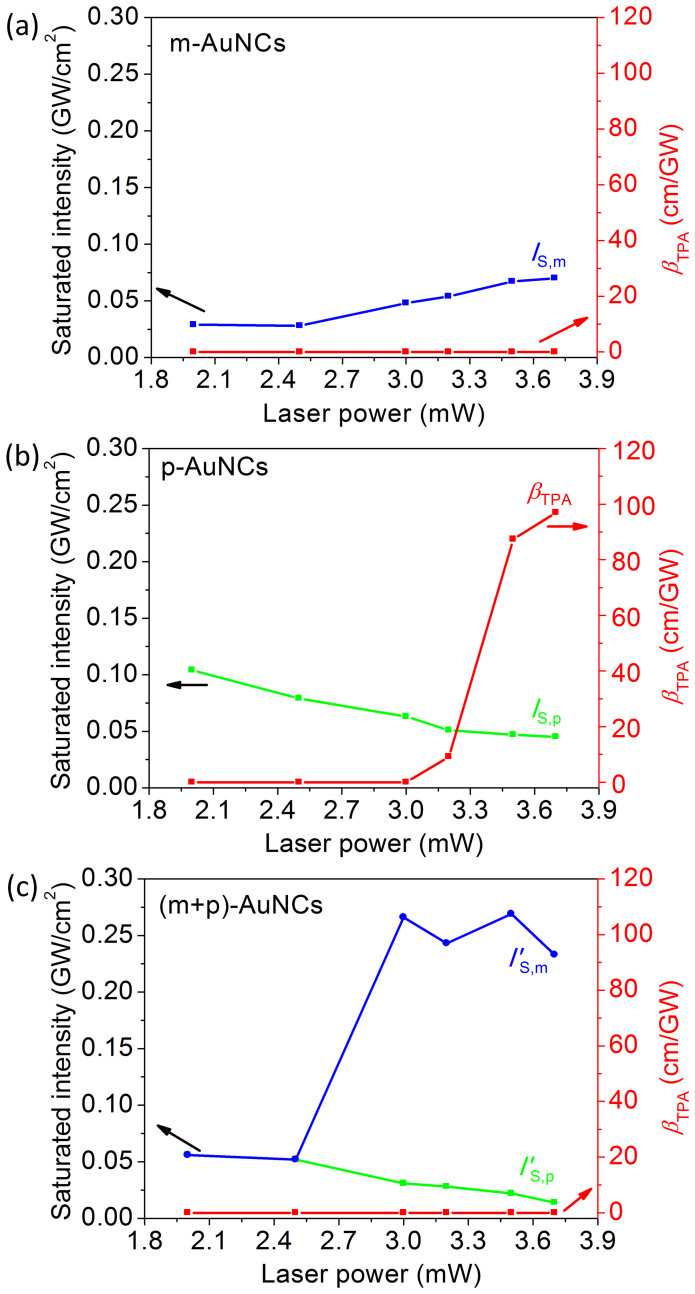
Saturable intensities (*I*_S,p_ and *I*_S,m_) and effective two-photon absorption coefficient (*β*_TPA_) as a function of input laser power (*P*). (a) Molecular-like AuNCs. (b) Plasmonic AuNCs. (c) Nanocomplex of plasmonic and molecular-like AuNCs. The energy transfer in the nanocomplex leads to a prominent increase in the saturable intensity *I*_S,m_ of the molecular-like AuNCs and decreases in the two-photon coefficient *β*_TPA_ and the saturable intensity *I*_S,p_ of the plasmonic AuNCs.

A pure saturable absorption is observed in the molecular-like AuNCs. As shown in Figure 2b, a peak near approximately *z* = 0 is demonstrated in the Z-scan nonlinear transmittance, and the peak height increases as the input laser power (*P*) increases. The intensity *I*(*z*) dependence of the saturable absorption is described as,
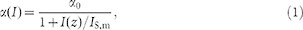
where *α*_0_ is the absorption coefficient in the weak field, and *I*_S,m_ is the saturable intensity of the molecular-like AuNCs. The intensity *I*(z) is related to the position *z* in the Z-scan measurements (Figure 2a) by
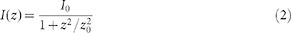
The nonlinear transmittance of the laser beam within the sample has a dependence on the propagation distance *z*′ described by
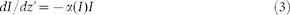
In an individual two-level system, the saturable intensity *I*_S,m_ is described by the relationship,
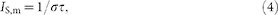
where *σ* is the absorption cross-section of the ground state, and *τ* is the lifetime of the excited state. A similar SA process is observed in the Au nanorod solutions reported by W. Ji's group and is due to the bleaching in the ground-state plasmon absorption induced by increasing laser intensity^2^. Extracted from the Z-scan nonlinear transmittance, the value of saturable intensity *I*_S,m_ of the molecular-like AuNCs increases from 0.029 GW/cm^2^ to 0.07 GW/cm^2^ as the input laser power increases from 2.0 mW to 3.7 mW (see Figure 3a). This large *I*_S,m_ is caused by a very small absorption cross-section of the AuNCs without plasmon resonance. Note that the saturable absorption will disappear (the corresponding *I*_S,m_ approaches infinity) when the size of the AuNCs further decreases to less than 1 nm^8^,^46^.

### Nonlinear Absorption of the Plasmonic AuNCs: SA→RSA

The SA**→**RSA nonlinear processes are observed in the plasmonic AuNCs, as shown in Figure 2c. The Z-scan nonlinear transmittance exhibits a pure peak at weak input laser power, but a dip appears in the peak (known as the M-shape) at strong laser power. This dip is caused by two-photon absorption processes. The power-dependent absorption of SA**→**RSA processes can be described by^4^,
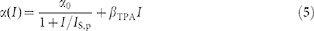
where *I*_S,p_ and *β*_TPA_ represent the saturated intensity and the effective two-photon absorption coefficient of plasmonic AuNCs, respectively. The measured *I*_S,p_ decreases from 0.104 GW/cm^2^ to 0.045 GW/cm^2^, and *β*_TPA_ increases from 0 cm/GW to 97 cm/GW as the input laser power increases from 2.0 mW to 3.7 mW (as shown in Figure 3b). The plasmonic AuNCs have a much smaller saturated intensity than that of the molecular-like AuNCs (*I*_S,p_ ≪ *I*_S,m_) owing to very efficient ground state absorption induced by plasmon resonance. The observed SA→RSA two-photon process in the plasmonic AuNCs is primarily attributed to the excited state absorption^2^,^4^, which is completely different from the RSA→SA processes caused by the saturation of two-photon absorption with notably strong excitation (280 GW/cm^2^) observed in the Au nanoparticle array^3^.

The strong dip observed in the Z-scan curves can be induced by several physical mechanisms, i.e., two-photon processes (including simultaneously absorption of two photons^8^,^57^ and excited state absorption^2^,^4^) and nonlinear scattering^56^,^58^,^59^,^60^,^61^. The effective TPA coefficient *β*_TPA_ of the plasmonic AuNCs measured at *I*_0_ = 0.211 GW/cm^2^ is approximately 97 cm/GW, which is very similar to the value reported by R. Philip with similar measurement conditions^8^. However, *β*_TPA_ is close to 0 (only SA is observed) when *I*_0_ < 0.171 GW/cm^2^ and significantly increases to 9.12 cm/GW as the *I*_0_ increases to 0.182 GW/cm^2^ (see Figure 3b and Figure S4). This power-dependent dip in Z-scan curves has been observed in both Au nanoparticles^2^,^57^ and semiconductor quantum dots^58^,^60^,^61^ and is assigned to the nonlinear scattering effect in the nanosystems. The power-dependent scattering from the plasmonic AuNCs is also observed (see Figure S5), and therefore, the strong dip in the Z-scan curves observed in plasmonic AuNCs could be attributed to the excited-state absorption of free electrons associated with nonlinear scattering^2^,^56^,^57^,^58^,^59^,^60^,^61^ (see Figures S4 and S5).

### Nonlinear Absorption of the Coupled Plasmonic and Molecular-Like AuNCs: SA→SA

Interestingly, two saturation processes of SA**→**SA (a narrow peak folded on a broad peak in the Z-scan trace) are observed in the nanocomplex of the plasmonic and molecular-like AuNCs (Figure 2d). As the power density increases (*z* → 0), the transmittance increases to ~60% at |*z*| ~ 2 mm and increases to ~80% at |*z*| ~ 0 mm (*P* = 3.5 mW). This nonlinear absorption of SA**→**SA processes can be approximately reproduced by the relationship,
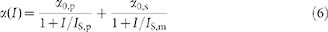
where *α*_0,p_ and *α*_0,m_ represent the linear absorption coefficients of the plasmonic and molecular-like AuNCs in the nanocomplex, respectively, and two corresponding saturated intensities *I*_S,p_ and *I*_S,m_ are modulated by the coupling of plasmonic and molecular-like AuNCs.

At weak laser power (*P* < 2.0 mW), only a single broad peak is observed in the Z-scan nonlinear transmittance, which is attributed primarily to the saturated absorption of the plasmonic AuNCs with a low-*I*_S,p_, and the contribution from the molecular-like AuNCs with a high-*I*_S,m_ can be neglected in this case. At strong laser power (*P* > 3.0 mW), Z-scan measurements demonstrate four interesting results: 1) The RSA processes caused by two-photon absorption of the plasmonic AuNCs are suppressed by the molecular-like ones; 2) A narrow peak folded on the centre of a broad peak is observed in the Z-scan traces, which is induced by SA→SA processes (a low-*I′*_S,p_ SA followed by a high-*I′*_S,m_ SA); 3) *I′*_S,m_ is measured as 0.233 GW/cm^2^ at *P* = 3.7 mW, which is an increase of approximately 233% compared with that of the bare molecular-like AuNCs; and 4) *I′*_S,p_ is measured as 0.014 GW/cm^2^ at *P* = 3.7 mW, which is a decrease of approximately 69% compared with that of the bare plasmonic AuNCs (Figure 3c).

**Figure 4:**
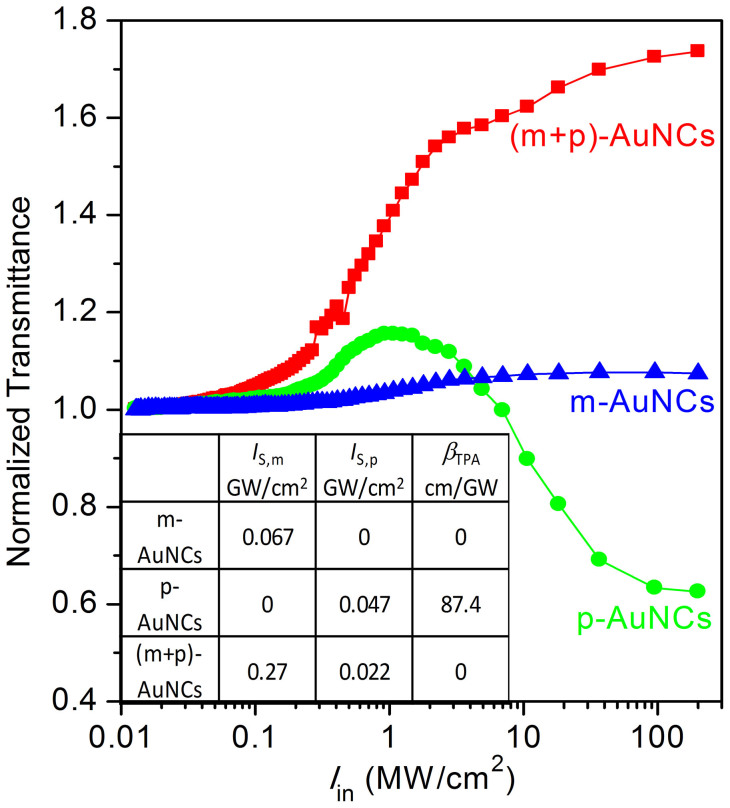
Measured power-dependent normalised transmittance of molecular-like and plasmonic AuNCs and the nanocomplex. Blue line: SA process of molecular-like AuNCs. Green line: SA→RSA processes of plasmonic AuNCs. Red line: SA→SA processes of the coupled plasmonic and molecular-like AuNCs. Inset: Measured nonlinear parameters of the individual AuNCs and the nanocomplex.

Figure 4 clearly demonstrates the power-dependent normalised transmittance of the plasmonic and molecular AuNCs and the nanocomplex. The molecular AuNCs have a SA with a saturated intensity of *I*_S,m_ = 0.067 GW/cm^2^. The plasmonic AuNCs exhibit SA→RSA processes with a saturated intensity of *I*_S,p_ = 0.047 GW/cm^2^ and an effective TPA coefficient of *β*_TPA_ = 87.4 cm/GW. The nanocomplex AuNCs demonstrate SA→SA processes with *I*_S,p_ = 0.022 GW/cm^2 ^and *I*_S,m_ = 0.27 GW/cm^2^.

### Population Rate Equations of the Coupled Plasmonic and Molecular-Like AuNCs

**Figure 5:**
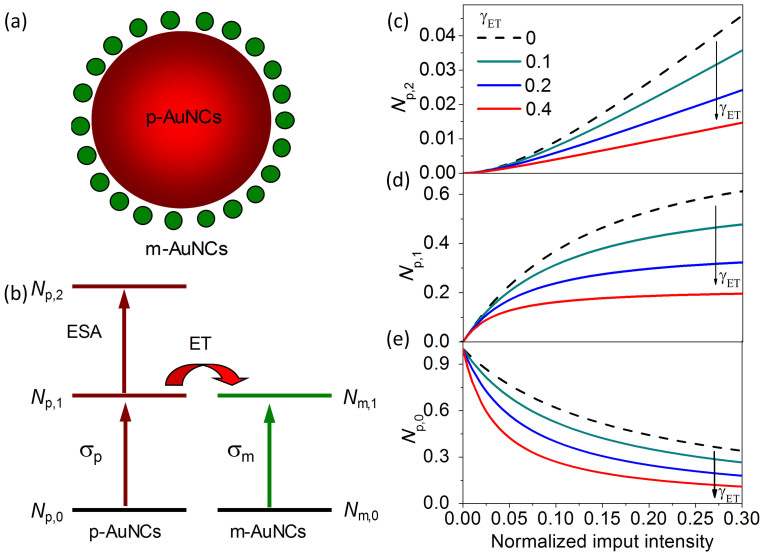
Illustrated nonlinear processes and calculated populations of the nanocomplex of plasmonic and molecular-like AuNCs. (a) Illustration of the molecular-like AuNCs absorbed on the plasmonic AuNCs. (b) Illustration of optical excitation and energy transfer in the nanocomplex. (c)–(e) Power-dependent populations on the two-photon level (*N*_P,2_), one-photon level (*N*_P,1_), and ground state (*N*_P,0_).

Finally, we discuss the physical mechanism of the enhanced nonlinear absorption of the nanocomplex consisting of plasmonic and molecular-like AuNCs. Figure 5a demonstrates that the small-sized molecular-like AuNCs are absorbed on the surface of the large-sized plasmonic AuNCs. Figure 5b illustrates two ground state absorptions (*σ*_p_ and *σ*_m_), an excited state absorption (*σ*_ESA_), and energy transfer (ET) in the nanocomplex of coupled plasmonic and molecular-like AuNCs. The population rate equations of the nanocomplex can be expressed by,
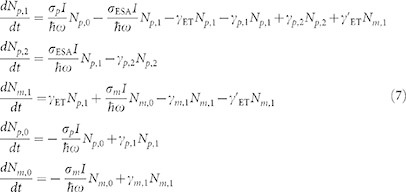
where *N*_p_ and *N*_m_ are the populations of the plasmonic and molecular-like AuNCs; the subscripts "0", "1", and "2" represent the ground state and the first and second excited states, respectively; *γ*_p,2_, *γ*_p,1_, and *γ*_m,1_ are the corresponding decay rates of the population relaxed to the lower level; and *γ*_ET_ is the energy transfer rate from the plasmonic AuNCs to the molecular-like AuNCs. In the SA→SA processes, the excited state absorption is efficiently suppressed and can be neglected, and thus, the two saturated intensities have the relationships,
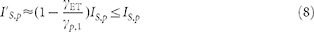

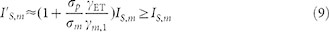


The populations of the five states of the nanocomplex of plasmonic and molecular-like AuNCs can be calculated from the equations in (7). Figure 5c–5e presents the power-dependent populations at the two-photon level (*N*_P,2_), one-photon level (*N*_P,1_), and ground state (*N*_P,0_) of the plasmonic AuNCs in the nanocomplex. As the energy transfer rate increases, the two-photon population *N*_P,2_ decreases (Figure 5c), both the saturation intensity and the population of the one-photon level decrease (Figure 5d), and the population bleaching effect of the ground state is prominently enhanced by energy transfer (Figure 5e). These calculations qualitatively coincide with the experimental observations of the suppressed TPA and enhanced saturated absorption of plasmonic AuNCs in the nanocomplex presented in Figures 2–4.

## Discussion

The rate equations deduced from this energy transfer model clearly reveal the following nonlinear behaviours: i) In the bare plasmonic AuNCs, the collaboration of *σ*_p_ and *σ*_ESA_ of the plasmon leads to a "W"-shaped Z-scan nonlinear transmittance (SA→RSA processes), and *I*_S,p_ ≪ *I*_S,m_ because *σ*_p_ ≫ *σ*_m_; and ii) In the nanocomplex of coupled plasmonic and molecular-like AuNCs, the energy transfer (*γ*_ET_) efficiently suppresses the excited state absorption (*σ*_ESA_) of plasmon, which eventually leads to the newly observed SA→SA nonlinear processes with 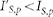 and 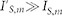 (Figures 3–5). Furthermore, these SA→SA processes can be optimised by adjusting the ratio of plasmonic and molecular-like AuNCs as well as their sizes.

In summary, the molecular-like AuNCs show a pure saturable absorption, whereas the plasmonic ones demonstrate a SA→RSA conversion. Intriguingly, the nanocomplex of the plasmonic and molecular-like AuNCs demonstrates dual saturable absorptions (SA→SA processes), which indicate that the two-photon RSA of the plasmonic AuNCs is suppressed and the one-photon SA of the molecular-like AuNCs is enhanced by efficient energy transfer in the nanocomplex. Our observations offer a strategy for improving the performance of plasmonic saturable absorbers and could find applications in such nonlinear optical nanodevices as liquid lasers and ultrafast modulators.

## Methods

### Sample Preparation

Chloroauric acid (HAuCl_4_·4H_2_O, 99.99%), silver nitrate (AgNO_3_, 99.8%), L-ascorbic acid (99.7%), hydrochloric acid (36–38%), and sodium borohydride (NaBH_4_, 96%) were all purchased from Sinopharm Chemical Reagent Co. Ltd. (Shanghai, China). Cetyltrimethylammonium-bromide (CTAB, 99.0%) was obtained from Amresco, Inc. All chemicals were used as received and without further purification. The water used in all reactions was obtained by filtering through a set of Millipore cartridges (Epure, Dubuque, IA).

The Au nanorods were prepared using a seed-mediated growth method^62^. The Au seed solution was formulated by adding 600 µL of ice-cooled NaBH_4_ solution into a 10 mL aqueous solution containing HAuCl_4_ and CTAB. For the synthesis of Au nanorods, 1.2 mL of aqueous HAuCl_4_ solution, 8 µL of aqueous AgNO_3_ solution, 7 µL of aqueous HCl solution, and 0.66 mL of aqueous ascorbic acid solution were mixed, followed by the addition of Au seed solution. The concentration of Au nanorods was estimated as approximately 8.0 nM according to the measured extinction coefficients at the localised surface plasmon resonance peak wavelength^63^. Subsequently, the Au nanorods were poured into a stainless steel autoclave and reshaped into Au nanospheres via annealing processes. Finally, the reactor was automatically cooled to room temperature. The resulting solution was centrifuged at 16,000 rpm to separate the supernatant and solids for further characterisation.

### Sample Characterisation

The TEM images and HRTEM images were measured with JEOL 2010 HT and JEOL 2010 FET TEM instruments at an acceleration voltage of 200 kV. The absorption spectra were collected on a TU-1810 UV-Vis-NIR spectrophotometer (Purkinje General Instrument Co. Ltd. Beijing, China).

### Z-Scan Measurements

The laser source (MPL 50 μj N532 9120510, Changchun New Industries Optoelectronics Tech. Co., Ltd.) for the Z-scan nonlinear transmittance measurements has a wavelength of 532 nm, a pulse width of 4.8 ns, and a repetition rate of 5000 Hz. The focus-length of the lens in the Z-scan setup is 150 mm.

## Supplementary Material

Supplementary Information Supplementary Information
