# Supplementary material for: Largely Enhanced Saturable Absorption of a Complex of Plasmonic and Molecular-Like Au Nanocrystals
Source: Sci Rep. 2015 Apr 15;5:9735. doi: 10.1038/srep09735 (PMC4398047; doi:10.1038/srep09735)
Supplement: Supplementary Information [file srep09735-s1.doc]

Supplementary Information for

“Largely Enhanced Saturable Absorption of a Complex of Plasmonic and Molecular-Like Au Nanocrystals”

*Si-Jing Ding^1^, Fan Nan^1^, Da-Jie Yang^1^, Xiao-Li Liu^1^, Ya-Lan Wang^1^, Li Zhou^1^, Zhong-Hua Hao^1^, Qu-Quan Wang^1,2^,**

** Corresponding author, E-mail:* [*qqwang@whu.edu.cn*](mailto:qqwang@whu.edu.cn)***

^1^ Department of Physics, Key Laboratory of Artificial Micro- and Nano-Structures of Ministry of Education, Wuhan University, Wuhan 430072, P. R. China

^2^ Institute for Advanced Studies, Wuhan University, Wuhan 430072, P. R. China


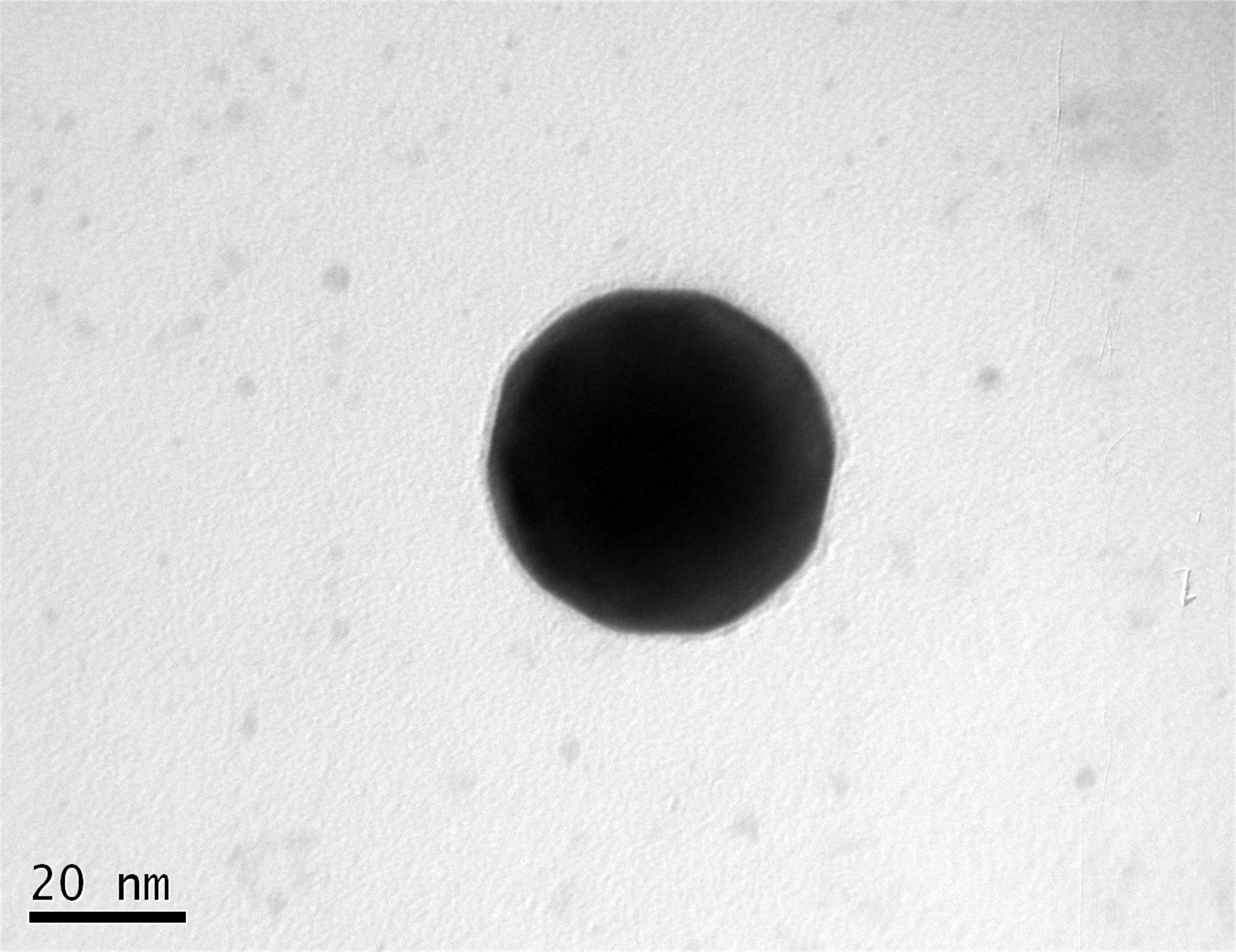


**Figure S1**. A TEM image of a large p-AuNC and a mass of small m-AuNCs. The small AuNCs are generated from the large one by thermal etching processes.


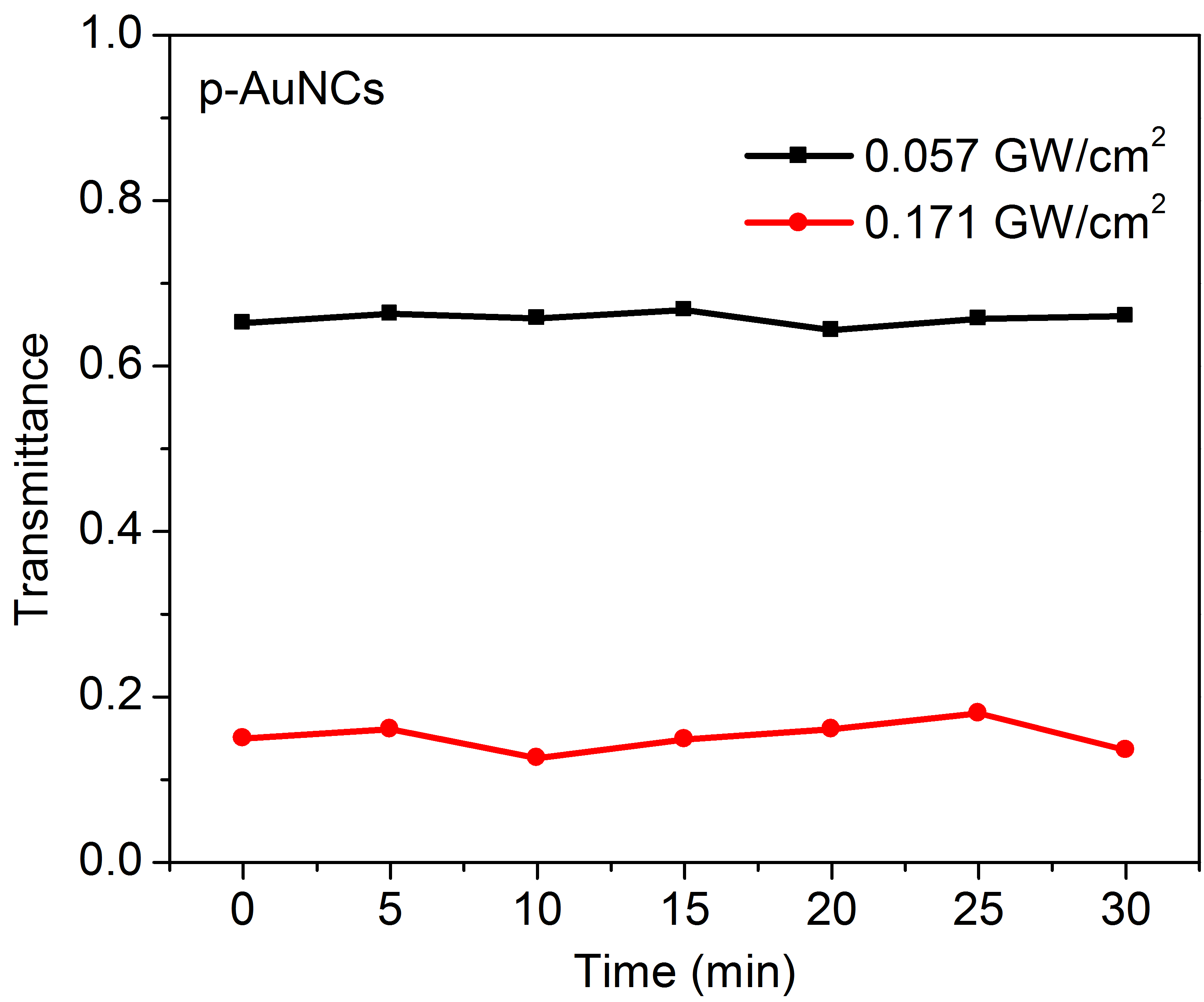


**Figure S2.** Time-dependent transmittance of the p-AuNC sample located at the Z-scan focus (the laser irradiance is set at 0.057 and 0.171 GW/cm^2^). The time-independent transmittance indicates the thermal fragmentation of p-AuNCs can be neglected.


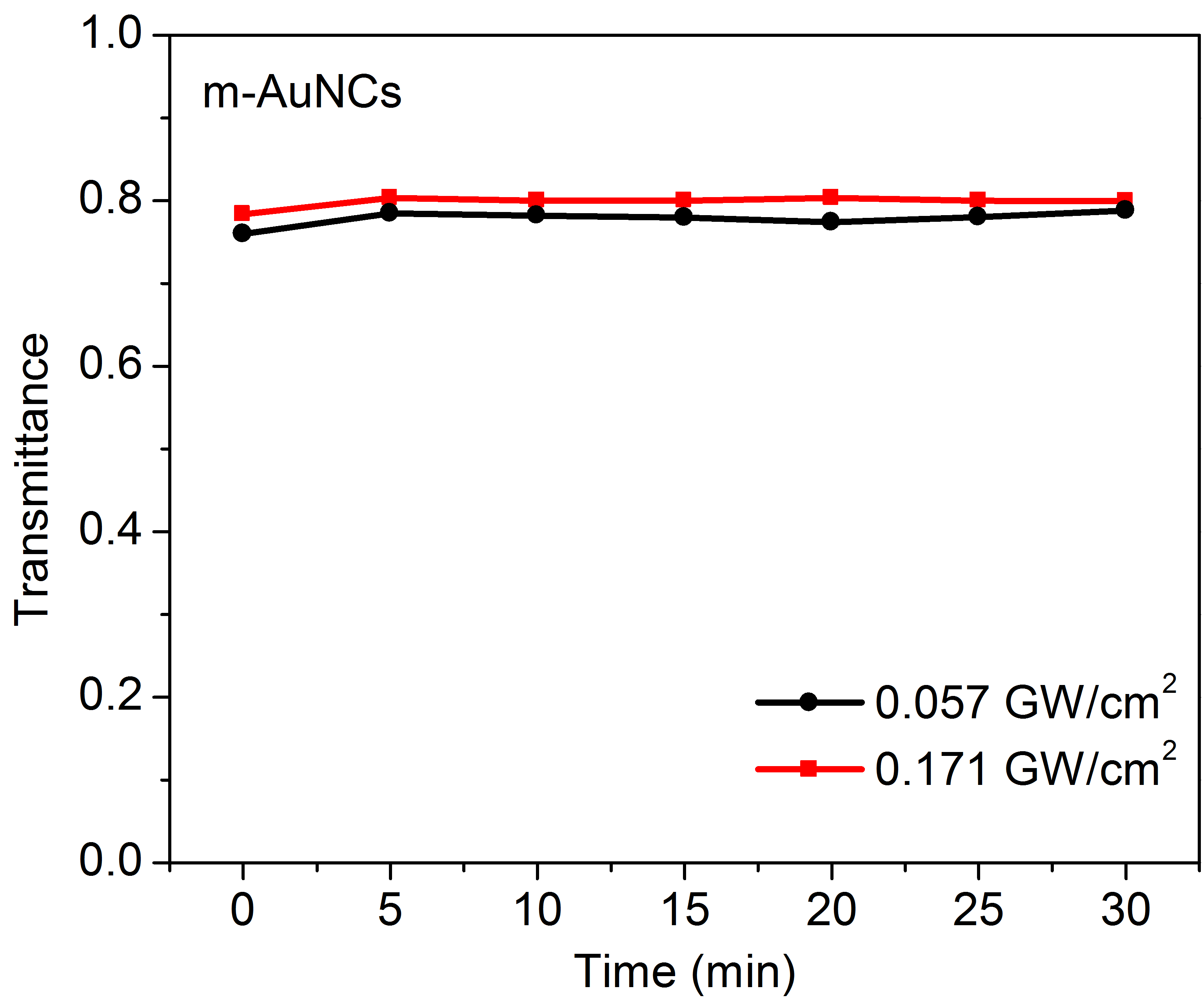


**Figure S3.** Time-dependent transmittance of the m-AuNCs sample located at the focus of Z-scan. The unchanged transmittance indicates well photo stability of small m-AuNCs at the laser irradiance 0.171 GW/cm^2^.


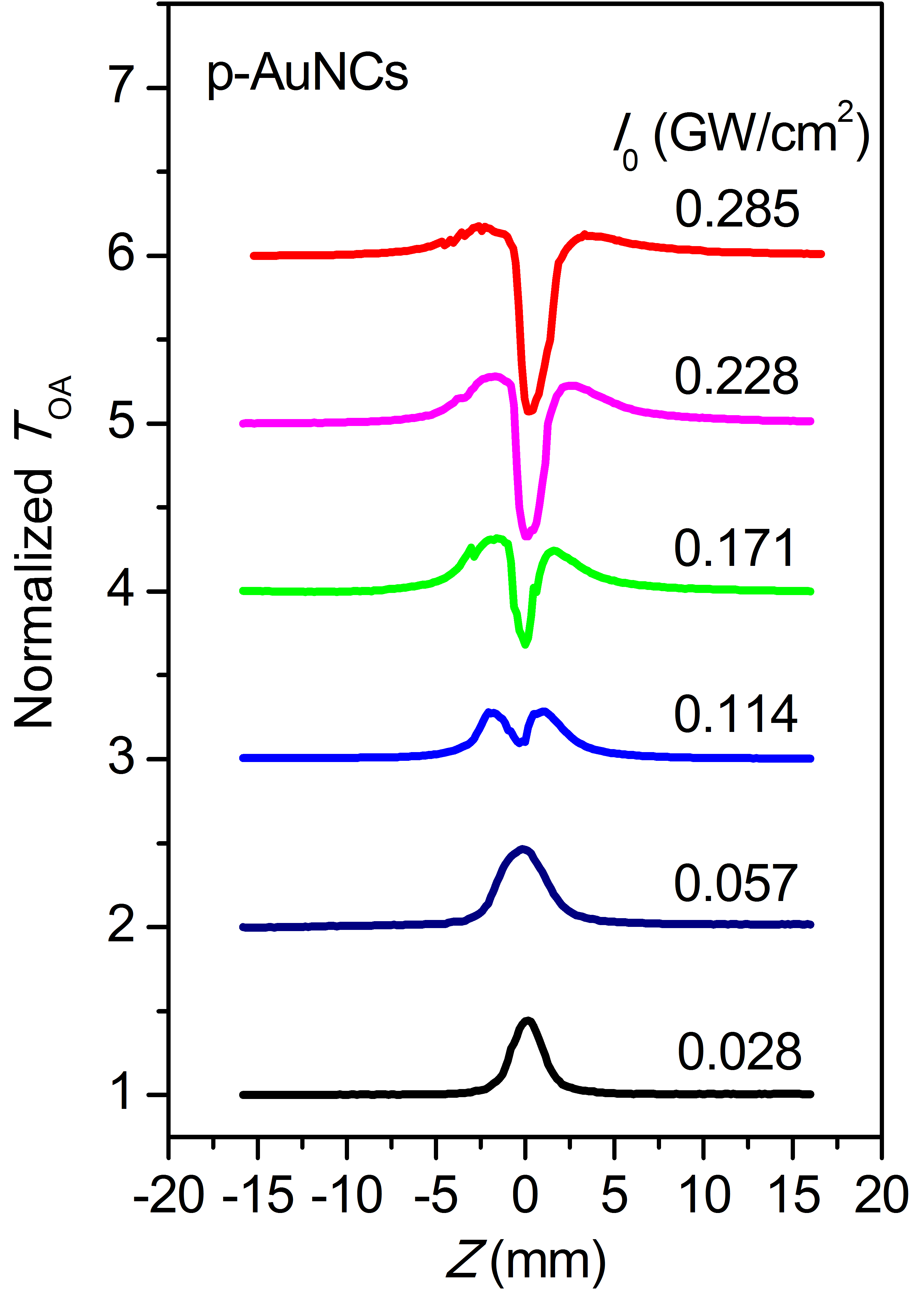


**Figure S4.** Power-dependent open-aperture Z-scan nonlinear transmittance of the plasmonic Au nanocrystals (p-AuNCs). SA is dominated at lower laser power < 0.057 GW/cm^2^, and RSA is dominated at higher laser power 0.171 GW/cm^2^.


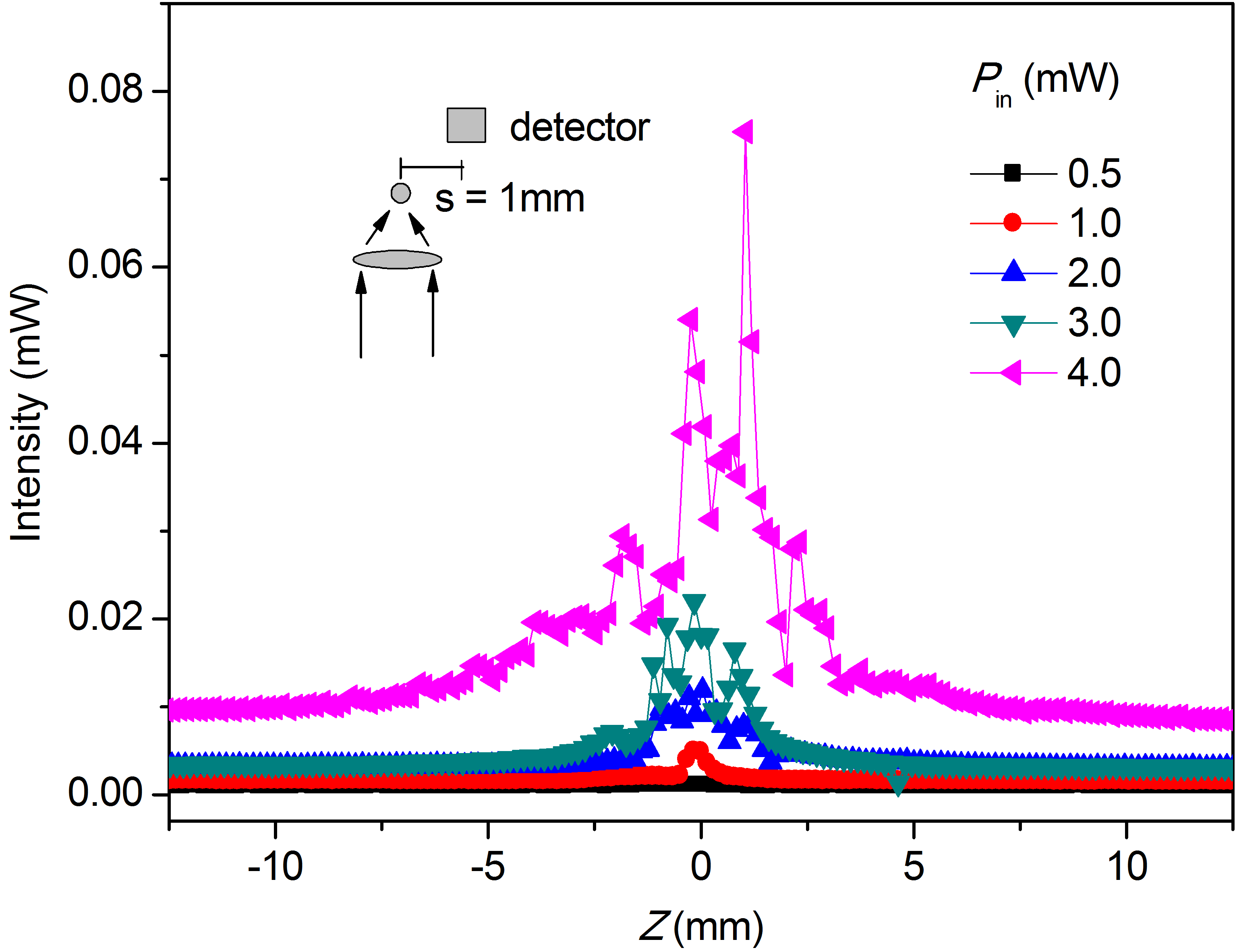


**Figure S5.** Power-dependent nonlinear scattering of the p-AuNCs suspensions measured by Z-scan setup with the input laser power *P*_in_ = 0.5, 1.0, 2.0, 3.0, and 4.0 mW. As *P*_in_ increases, the scattering nonlinearly increases; the peak of Z-scan curve of scattering is significantly broadened. Notice that the peak width the scattering Z-scan curve is prominently larger than the valley width of the transmittance Z-scan curve, the overall normalized scattering intensity peak is much less than the deep depth of RSA presented in Figure S4. This indicates that the strong deep in Z-scan transmittance of p-AuNCs is attributed to TPA associated with nonlinear scattering.


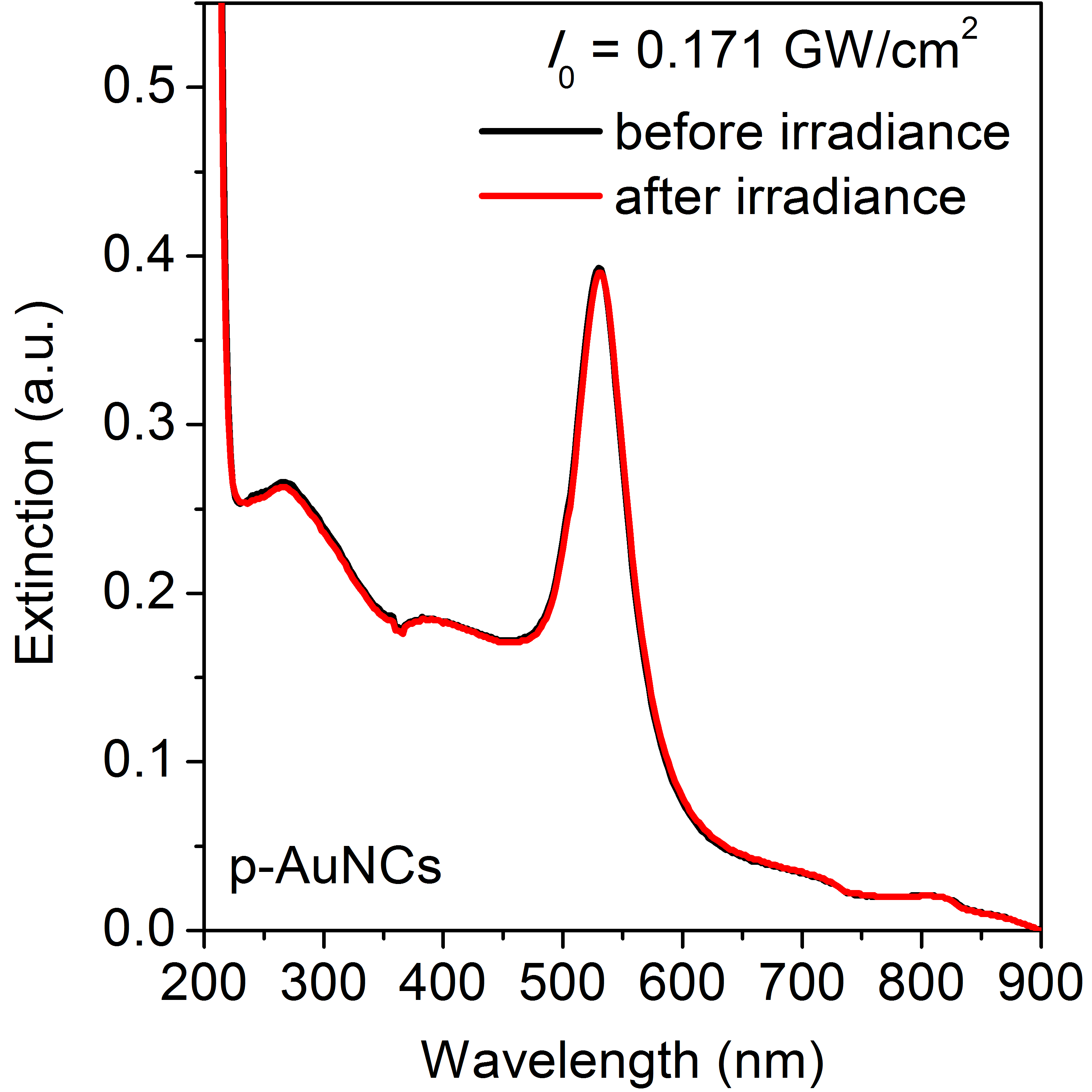


**Figure S6.** The absorption spectrum of the p-AuNCs sample on the Z-scan focus for two hours with laser irradiance 0.171 GW/cm^2^. The spectrum did not exhibit prominent variation before and after laser irradiance at 0.171 GW/cm^2^, which indicates that the degradation and reshape of the plasmonic AuNCs could be neglected at the low irradiance of 0.171 GW/cm^2^.


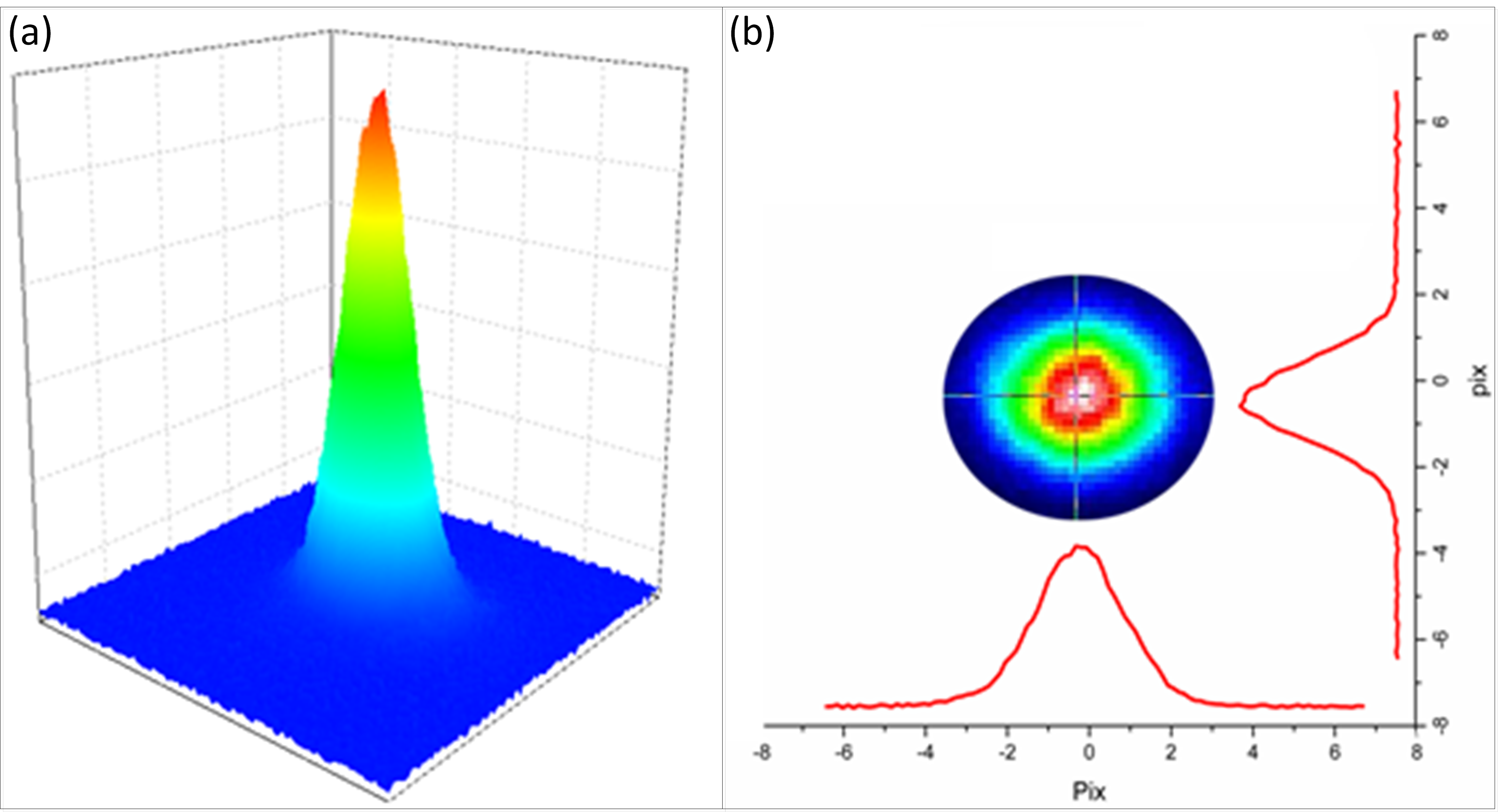


**Figure S7.** Spatial profile of the nanosecond laser used in Z-scan measurements. A Guassian spatial distribution is demonstrated.

**
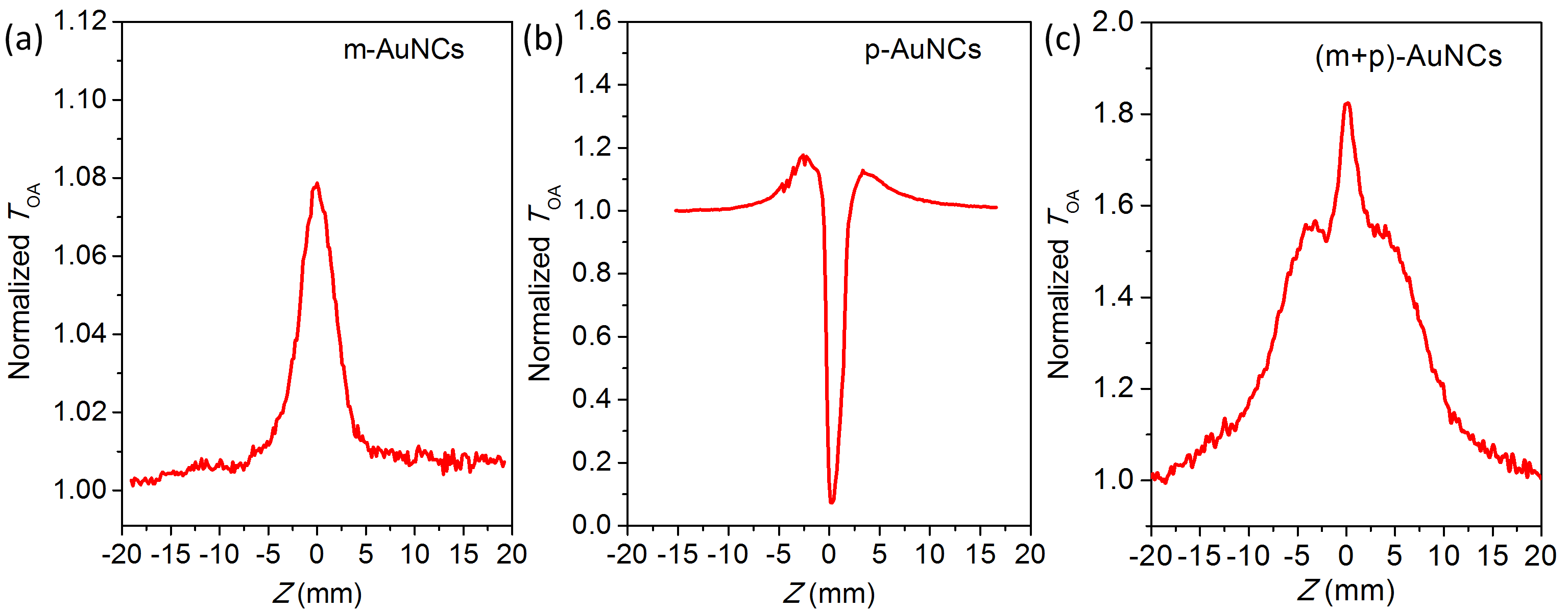
**

**Figure S8.** Three typical types of Z-scan curves of nonlinear transmittance. (a) SA process of m-AuNCs described by *α*(*I*) = *α*_0_/(1+*I*/*I*_S,m_). (b) SA→RSA processes of p-AuNCs described by *α*(*I*) = *α*_0_/(1+*I*/*I*_S,p_)+*β*_TPA_*I*. (c) SA→SA processes of (m+p)- AuNCs complex described by *α*(*I*) = *α*_0,p_/(1+*I*/*I*_S,p_)+ *α*_0,m_/(1+*I*/*I*_S,m_).
